# Longitudinal Associations Between Pain Trajectories and Cognitive Function: The Role of Neighborhood Context

**DOI:** 10.1093/geroni/igaf122.2296

**Published:** 2025-12-31

**Authors:** Eunbea Kim, Jeong Eun Lee, Peter Martin

**Affiliations:** Auburn University, Auburn, Alabama, United States; Iowa State University, Ames, Iowa, United States; Iowa State University, Ames, Iowa, United States

## Abstract

Physical pain is a prevalent issue among older adults and a potential factor exacerbating age-related cognitive impairment. However, the longitudinal relationship between pain and cognitive function has yet to be fully accomplished. While previous research has focused on the direct impact of pain on cognitive outcomes, less attention has been given to the role of social and environmental contexts that may moderate cognitive decline associated with pain. This study examines the association pain trajectories and cognitive function and explores whether neighborhood characteristics (neighborhood social cohesion and physical environment) moderate this relationship. Using data from the Health and Retirement Study (HRS), we analyzed a sample of adults aged 50 and older (n = 6,983, Mage = 44.92) across 16 years. Growth mixture modeling identified groups of pain trajectories and examined their relationships with cognitive function. This study also tested the interaction effect between pain trajectories and neighborhood characteristics on cognitive function. The results indicated that individuals with persistent moderate- and severe- levels of pain exhibited lower cognitive function compared to those with mild level of pain over time. While neighborhood characteristics did not significantly moderate this relationship, group-specific regression revealed that higher neighborhood social cohesion and lower neighborhood physical disorder were related to higher level of cognitive function among individuals with mild pain trajectory. These findings suggest that social context may play a protective role in cognitive function when pain levels are low but may have limited impact in cases of severe pain, where cognitive decline may already be progressing.

